# The complete chloroplast genome sequence of *Bienertia sinuspersici*

**DOI:** 10.1080/23802359.2016.1174083

**Published:** 2016-06-20

**Authors:** Beomsoo Kim, Jingyu Kim, Hyun Park, Joonho Park

**Affiliations:** a Department of Fine Chemistry, Seoul National University of Science and Technology, Seoul, Korea;; b Korea Polar Research Institute, Incheon, South Korea

**Keywords:** *Bienertia sinuspersici*, C4 photosynthesis, single cell

## Abstract

The *Bienertia sinuspersici* chloroplast whole genome (cpDNA) sequencing was completed in this study. *Bienertia sinuspersici* chloroplast genome is 153,472 bp in length and contains 127 genes, such as 83 unique protein-coding genes, 36 tRNA genes and eight rRNA genes. There were two inverted repeat regions (IR) and small and large single-copy regions (SSC and LSC) with 24,948 bp, 19,016 and 84,560 bp, respectively. 59% of the *B. sinuspersici* cpDNA consisted of gene-coding regions (protein-coding and RNA genes). The overall GC contents of the *B. sinuspersici* cpDNA were 36.59% and in the LSC, SSC and IR regions were 34.47%, 29.42% and 42.94%, respectively. A phylogenetic analysis of seven complete cpDNA from Chenopodiaceae family shows that *B. sinuspersici* cpDNA is closely related to *Salicornia* species.

Chloroplasts are the essential organelles in plant cells. Plants contain own genetic system for the photosynthesis. Chloroplast DNA of higher plants contains about 150 kb. Angiosperm cpDNAs have a 20–30 kb IR that encodes a complete set of rRNA, mRNA genes and tRNA, and have similar order of genes and sequences (Palmer & Stein 1986). Complete chloroplast genome sequences have been used for plant identification and phylogenetic studies (Gielly & Taberlet 1994; Moore et al. 2007).

The efficiency of photosynthetic carbon fixation in plants has significant limitations due to the oxygenase activity of the enzyme Rubisco, especially environment in warmer temperatures or water stress. In order to minimize oxygenase activity, plants adopted novel photosynthesis mechanisms called the C4 photosynthesis (Ehleringer et al. 1997; Edwards et al. 2004; Edwards & Voznesenskaya 2011).

The Chenopodiaceae family contains about 1300 species and many species conduct C4 photosynthesis. Traditionally, it was believed that C4 plants strictly separated into different cell types for photosynthesis which is Kranz C4. But in some species, it is found to have novel mechanisms for C4 photosynthesis by compartmentalization of chloroplast and other organelles in the single cell which is called single cell C4. There were complete chloroplast genomes for Kranz C4 plants but not for single cell C4 plants (Eckardt 2006; Stutz et al. 2014). The genomic database for single cell C4 photosynthesis will be foundation to reveal single cell C4 developing mechanisms. Seeds of *B. sinuspersici* were provided by Dr. Offermann, Leibniz-Universitaet Hannover in Germany. In this study, we reported the complete chloroplast genome of the *B. sinuspersici* which is Chenopodiaceae family angiosperm plant with single cell C4 system. It is from main range around the Persian Gulf countries and the northern side of the Gulf of Oman (Akhani et al. 2005). This cpDNA obtained through Illumina sequencing system (Illumina Inc., San Diego, CA). The chloroplast DNA of *B. sinuspersici* is double-stranded circular DNA with 153,475 bp in length. Its structure is similar with general chloroplast genomes from the higher plants. Chloroplast genome of *B. sinuspersici* composed of two IR regions of 24,949 bp, which was divided by a LSC region of 84,561 bp and a SSC region of 19,016 bp. The overall GC contents of the *B. sinuspersici* cpDNA were 36.59% and in the LSC, SSC and IR regions were 34.47%, 29.42% and 42.94%, respectively. The entire gene content and positions of 127 individual genes (83 protein encoding genes, 36 tRNA genes, 8 rRNA genes), 17 genes (*rpl2, rpl23, trnI-CAU, ycf2, trnL-CAA, ndhB, rps7, rps12, trnV-GAC, rrn16, trnI-GAU, trnA-UGC, rrn23, rrn4.5, rrn5, trnR-ACG* and *trnN-GUU*) are duplicated in the IR regions. Fourteen genes (*rps16, atpF, rpoC1, petD, rpl16, ndhB, ndhA, ndhB, trnL-UAA, trnV-UAC, trnI-GAU, trnA-UGC, trnA-UGC* and *trnI-CAU*) contained one intron, while two genes (*clpP* and *ycf3*) had two introns. The complete chloroplast genome sequence was submitted to GenBank under the accession no. KU726550.

A phylogenetic tree was constructed by complete chloroplast genome of Chenopodiaceae family using Muscle to align multiple sequences and MEGA7 (MEGA Inc., Englewood, NJ) to construct a maximum-likelihood tree. *Salicornia* genus was closer neighbors of *B. sinuspersici* than other species (Figure 1).

**Figure 1.**
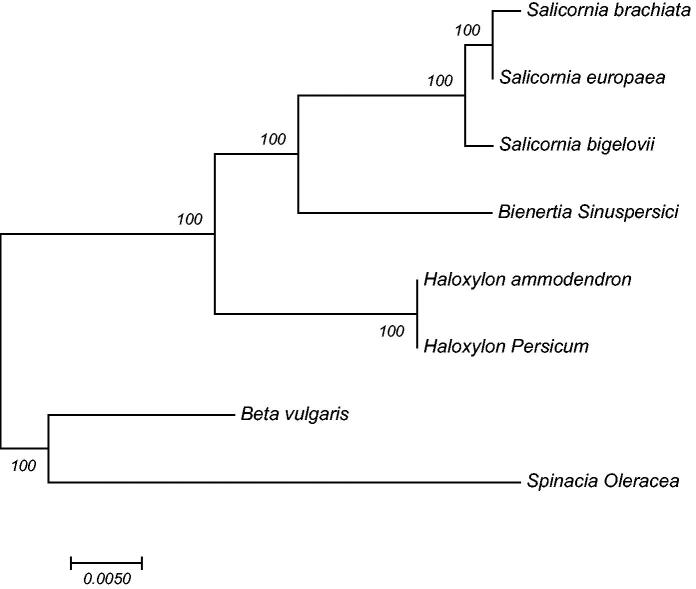
Molecular phylogeny of Chenopodiaceae family using chloroplast genome. The phylogenetic tree is constructed by maximum-likelihood method with 1000 bootstrap replicates. GenBank accession numbers is listed as the following: NC_027224.1, NC_027225, NC_027226.1, KU726550, KF534478, KF534479, KJ081864 and AJ400848.1.
